# Work-Family Conflict among Iranian Emergency Medical Technicians and Its Relationship with Time Management Skills: A Descriptive Study

**DOI:** 10.1155/2020/7452697

**Published:** 2020-05-11

**Authors:** Mehdi Beyramijam, Yousof Akbari Shahrestanaki, Hamidreza Khankeh, Mohsen Aminizadeh, Ali Dehghani, Mohammad Ali Hosseini

**Affiliations:** ^1^Health in Emergency and Disaster Research Center, University of Social Welfare and Rehabilitation Sciences, Tehran, Iran; ^2^Emergency Department, School of Paramedicine, Qazvin University of Medical Sciences, Qazvin, Iran; ^3^Department of Clinical Science and Education, Karolinska Institutet, Stockholm, Sweden; ^4^Health in Emergency and Disaster Research Center, Kerman University of Medical Sciences, Kerman, Iran; ^5^Department of Nursing, School of Nursing and Paramedical, Jahrom University of Medical Science, Jahrom, Iran; ^6^Department of Nursing, University of Social Welfare and Rehabilitation Sciences, Tehran, Iran

## Abstract

**Background:**

Work-Family Conflict (WFC) is a form of interrole conflict in which an active participation in occupational activities causes strain and interferes with family roles of workers and vice versa. It is a major source of occupational stress among workers and personnel. Emergency Medical Technicians (EMTs) are an important part of the healthcare system that respond to emergencies. The EMTs experience high level of job stress, which may affect their ability to perform their family roles, and, on the other hand, actively performing their family responsibilities may interfere with the effective delivery of the already stressful activities at workplace.

**Objective:**

The aim of this study was to determine the prevalence of WFC among Emergency Medical Technicians in Iran and its relationship with time management skills.

**Methods:**

This was a descriptive study. In this study, 271 EMTs from the western part of Iran completed the questionnaire for the assessment of WFC. The Carlson Family-Conflict Questionnaire and the “Time Management Behaviors Scale” developed by Macan were used as evaluation instruments. The data were analyzed by SPSS software version 16. Appropriate statistical analysis such as mean and standard deviation, Pearson correlation, and Spearman rank correlation was applied for analyzing the data in SPSS.

**Results:**

The majority of the participants reported some degrees of WFC. Statistical analysis showed a significant inverse correlation between total WFC score and total “Time Management Behaviors scale” score (*r* = −0/381، *p* < 0/0001). In the present study, there was no significant correlation between total WFC score and demographic factors such as educational level, age, sex, marital status, number of family members, need for family member care, and work experience (*p* > 0.05).

**Conclusions:**

The findings of this study indicate that time management behaviors and skills can reduce WFC among Emergency Medical Technicians. Therefore, it is recommended that prehospital emergency authorities and policymakers plan and implement measures such as reducing the duration of shift-work schedules, decreasing shift-change restrictions, and organizing regular time management courses. Also, employment of local inhabitants is preferred to geographically distant individuals with similar qualification as this will reduce the distance between home and workplace.

## 1. Background

Work-Family Conflict (WFC) is an important indicator of healthy lifestyle and job satisfaction among employees, and it is often associated with potential health and socioeconomic consequences [1–5]. Over the past few years, family life has undergone drastic changes. Increase in family expenditure has caused more family members to join the labor force, including mothers. On the other hand, there is a rise in single-parent households, and the single parent is required to meet job schedules and at the same time manage his/her home. There are also many employees who take care of the elderly in their families [6] along with their work schedules. All these have contributed to an increase in the prevalence of WFC. Evidence indicates that cultural, political, economic, and social factors have influence on labor division and role expectations in work and family in different communities [7]. Despite extensive studies that have been conducted on the prevalence and attributes of Work-Family Conflict elsewhere in the world [8–10], this field has not gained much attention in the Middle East countries, especially in Iran. The sociocultural practices of the people in the Middle East are somehow similar, and, therefore, it can be expected that some aspects of WFC are similar in the region. Recently, Eshak and colleagues conducted some studies in this field in one of the Middle East countries [4, 5, 11]. They reported that the prevalence of WFC is moderate .According to Greenhouse and Beutell, WFC occur in situations where there is a bilateral incompatibility between work and family responsibilities, such that family life interferes with work life and work life interferes with family life [12]. Healthcare professionals, specifically Emergency Medical Technicians (EMTs), are exposed to high level of job stress. They work as frontline healthcare workers in the public health systems and have a difficult and stressful job status. EMTs are present for a long time at their workplaces, and they work in harsh weather conditions (rainy, heat, cold, storm, and blizzard weathers), are exposed to violence, and work on vacations and public holidays [13, 14].

The family members of EMTs live through such conditions and although they often are aware of the work schedules of the working family member, they are rarely sure when he/she will return from work [14]. Many studies have been conducted on WFC among other healthcare professionals such as nurses and physicians [15–18]. But these studies are very limited among Emergency Medical Technicians, especially in Iran. Studies on emergency medical service workers have indicated that increase in time and duration of shift-work lead to an increase in occupational injuries and illnesses [19].

The duration of shift work among Iranian emergency medical personnel is 24/48 (24 hours on site and 48 hours off) or 48/24 (48 hours on site and 24 hours off) or a combination of both, which is not compatible with the Occupational Safety and Health Administration (OSHA) standards. According to the OSHA standards, the employees must not work more than 8 consecutive hours for 5 days a week and they require at least an 8-hour break between shifts [20].

The busy work schedule can have an immense effect on WFC. Therefore, there is a need to conduct a study on the prevalence of WFC among Emergency Medical Technicians and its related factors in Iran. Studies have shown that empowerment, efficient time management, decision-making, and problem solving skills reduce the rate of WFC [21]. According to the study of Adams and Jex (1999), among the aforementioned factors, time management skills play a more effective role in reducing WFC [22]. Accordingly, this study was conducted to determine the rate of Work-Family Conflict among Emergency Medical Technicians in Iran and its relationship with their time management skills.

## 2. Methods

In this descriptive study, all Emergency Medical Technicians in Hamadan and Qazvin provinces (from western part of Iran) were selected by the census method and questionnaires were sent to their workplace. The Iranian version of Work-Family Conflict Questionnaire [1] was used to determine the rate of WFC among the Emergency Medical Technicians. This tool [23] was translated into Persian by Rasouli et al. with acceptable validity and reliability [24].

The reliability of the subscales of this tool was reported by Karlson et al. as between 76% and 89% [23]. This questionnaire includes 18 questions and 6 dimensions including time-based Work-Family Conflict (Questions 1–3), time-based Family-Work Conflict (Questions 4–6), Work-Family Conflict based on frictional power (Questions 7–9), Family-Work Conflict based on frictional power (Questions 10–12), Work-Family Conflict based on behavior (Questions 13–15), and Family-Work Conflict based on behavior (Questions 16–18). The questions of this tool are answered on a six-point Likert scale (from strongly disagree to strongly agree).

The “Time Management Behaviors Scale” developed by Macan (1990) was used to determine time management skills among the EMTs [25]. It includes 39 questions that assess common concepts of time management behaviors. Its subscales consist of answers to “management of meetings” questions (Questions 16, 34, 36, and 37), “communication” (Questions 8, 15, 14, 17, 32, 33, and 35), “operational planning” (Questions 2, 7, 6, 10, 22, 25, 23, and 26), “goal prioritizing” (Questions 4, 6, 18, 28, 30, and 38), “goal setting” (Questions 1, 3, 5, 29, and 39) and “delegation of authority” (Questions 11, 12, 13, 21, 31, and 24).

In different studies, the reliability of this tool has been estimated to be between 50% and 90% using Cronbach's alpha coefficient [25]. Also in the study of Rasooli et al., the reliability of this tool was reported as 75% using Cronbach's alpha coefficient [24]. On a Likert scale, there are 5 possible responds to the questions of this tool: rarely, sometimes, most of the time, usually, and very often. A demographic questionnaire was used to collect data of the demographic characteristics of the participants including age, gender, marital status, shift-working schedule, number of hours of work required per month, work history, records, and number of family members.

The study was approved by Students' Research Committee of the University of Social Welfare and Rehabilitation Sciences, and the required permissions were obtained from the Emergency Medical Services and Emergency Management Centers of Hamadan and Qazvin provinces. Finally, the data were analyzed using appropriate statistical analysis such as mean and standard deviation, Pearson correlation, and Spearman rank correlation in SPSS IBM16.

## 3. Results

Out of the 350 questionnaires distributed, 271 questionnaires were filled by the participants, representing a response rate of 66%. Most participants were males (97%) and married (69%). The mean age of the participants was 31 ± 6 years. Most of them (56%) had a family with at least two members. Most of them were holders of associate degrees (45%) and bachelor degrees (33%). Among the participants, 22% (43 individuals) were also enrolled in various-degree programs, including associate degrees (6.3%), bachelors (8.3%), masters (6.8%), and doctorates (0.5%).

In the present study, majority of the participants (68%) reported variable shift-work schedule; 24/48 (24-hour on site and 48-hour off) and 48/24 (48-hour on site and 24-hour off), and most of them (63.5%) reported “shift exchange restriction.” “Overtime working” was reported by 92% of the respondents of the questionnaire. In order to meet their family expenditure, 38.5% of the EMTs reported working in rural EMS bases in addition to urban EMS bases. Almost half of the participants (49%) reported a distance of 20–40 km between home and workplace. 51% of the participants stated that at least one member of their family needed ongoing daily care, and most of the participants (80%) had not participated in any training course on time management skills at the time of the study (Table 1).

The rate of WFC was moderately high among the EMTs (55.11 ± 81.45 (Table 2)). Pearson correlation analysis showed a significant inverse relationship between the total score of WFC and total score of time management skills (*p*=0.0001, *R* = −0/381) (Table 3). Also, there was a significant inverse relationship between total WFC score and time management domains, such as “goal setting,” “goal prioritizing,” “operational planning,” and “delegation of authority.” Nonetheless, there was no statistically significant relationship between total WFC score and “communication management” and “meeting management” domains (Table 4).

According to the results of one-way analysis of variance (ANOVA), there was no significant relationship between total WFC score and personnel demographic factors such as education level, age and sex, marital status, number of family members, need for family members care (need for care), economic status, and work experience (*p* > 0.05). However, the rate of WFC was higher in the employees with poor economic status (61.26 ± 7.96) and in those who had at least one dependent in their family who needed daily care (56.87 ± 75.10) as compared to other employees.

Also, there was a significant relationship between the rate of WFC and occupational factors such as “work-shift schedule” and “shift exchange restriction” and “employment status” (*p* > 0.05) (Table 5). This relationship was not significant for factors such as “ working hours per month,” “workplace location” (“rural EMS base,” “urban EMS base,” etc.), and “the distance between home and workplace.”

## 4. Discussion

The main aim of this study was to determine the prevalence of Work-Family Conflict among Emergency Medical Technicians in Iran. In the present study, the prevalence of WFC among Emergency Medical Technicians was found to be moderately high. This result is justified by the stressful occupational conditions of healthcare workers, including EMTs, nurses, and physicians. Mache (2015) indicated in his study that physicians working in German hospitals suffer high WFC [26].

The degree of WFC depends on various factors such as personal, family, and occupational circumstance [27]. Contrary to the findings of the present study, Rittippant et al. [27], Taylor et al. [7, 28], Butler et al. [29], Boyar et al. [30], and Punyasiri et al. [31] reported that the extent of WFC correlate with individual factors (i.e., age, sex, and education), family factors (i.e., number of family members and number of dependents in the family), and occupational factors (i.e., “workload,” “number of hours worked per week,” “shift-work schedule,” “irregularities at work schedule,” and “lack of flexibility in the work plan”). In the present study, there was no significant correlation between WFC and the individual factors (including educational level, age, sex, and marital status), family factors (including number of family members and the presence of family member who needed daily care), and occupational factors (such as work history, number of work hours per month, workplace (rural EMS base or urban EMS base), distance between workplace and home, and the average number of missions per day (workload) of the EMTs).

In other studies [11, 32], household income and social support from family were the two most important factors that influence WFC. However, in the present study, there was no relationship between WFC and household income. Individual factors such “education level,” “marital status,” “number of family members,” and the “presence of a family member who needed daily care” directly related to financial and social support.

Also, this relationship was not significant for “workplace” (urban or rural EMS base) and “the distance between workplace and home.” However, the rate of WFC was higher in rural-based EMS staff (58.21 ± 11.28) compared to urban-based EMS staff and , as well, the rate was higher in the EMTs who reported a distance of more than 60 km between home and workplace (58.12 ± 16.37). Most of the EMTs working in rural emergency bases reported that the bases are located in areas far from cities where they live. The high rate of WFC in these employees could be explained by the limited amount of time spent with the family and the long distance between workplace and home. A study by Arif et al. reported that long working hours (being away from the family) not only had an impact on one's health, but also had a negative effect on “family health” [33].

In this study, occupational factors such as “work-shift schedule,” “employment status,” and “shift exchange restriction” had a significant relationship with WFC (Table 3). WFC was higher in the EMTs with 24-hour shifts (24 h on site and 48 h off) compared with EMTS with only morning shift (6 days a week) and EMTs with less than 24-hour shifts (12-hour shifts). In the present study, we found that restrictions on shift-work change increase the rate of WFC. In this regard, the studies of Kelly and Huang [34] and Roeters et al. [35] showed that employees who had more control over change of their work schedules experienced less WFC. Sleep disorders may be one of the causes of the increase of WFC in EMTs. The study of Eshak et al. (2019) demonstrated that Work-to-Family Conflict (WFC) and Family-to-Work Conflict (FWC) were associated with sleep disorders. Also, a study conducted by Jacobsen et al. reported that higher levels of WFC were significantly associated with sleep disorders, including deficiency, short sleep duration, and perceived sleep insufficiency, in patient care workers [16].

The findings of this study indicate that employees with a “contractual” job status are more likely to experience WFC than employees with “formal” job status. This seems likely to be related to a higher occupational security in formal staff compared to contracted staff. According to the instructions of the Governmental Human Resources Unit in Iran, contracted staff have lower job security than official staff. This finding is consistent with the findings of the study of Taylor et al. (2009). They found a significant relationship between occupational security and Work-Family Conflict [28].

In line with the main hypothesis of this study, we found that there is a negative (inverse) relationship between WFC in EMTs and time management skills. This means that the application of time management skills such as “goal setting,” “goal prioritizing,” “operational planning,” and “delegation of authority” decreases the level of WFC. This finding is consistent with the findings of Rasooli et al. [24], Claessens [25], and Adams and Jex [22]. Rasouli et al. reported that training programs aimed at improving time management skills had positive effects on WFC. As well, Glassens (2004) and Adams (1999) reported a similar finding.

## 5. Limitations and Future Directions

The data used in the present study were correlational; therefore, the causal direction of the observed relationships cannot be determined. It is recommended that a qualitative study be conducted to deeply investigate the risk factors of WFC among Iranian EMTs. Also, with respect to the factors aforementioned in the discussion section, a study aimed at investigating the probable association between WFC and sleep disorders among Iranian EMTs could be valuable. Currently, in Iran, usually males are employed in prehospital emergency service centers; hence majority of the participants in the present study were males and the results cannot be reasonably generalized for both male and female EMTs. It would be useful to perform a similar study in female technicians.

## 6. Conclusion

The results of this study showed that Emergency Medical Technicians suffer from high WFC and it can be a source of stress and mental pressure for themselves and their families. Prehospital emergency authorities and policymakers can with planning and implementing the measures such as reducing the duration of shift-work schedules (24- or 48-hour shift-work), execution of regular time management courses, and decrease in shift-change restriction, as well as reduce in distance of workplace and home by employing native staff, reduce the WFC in emergency technicians.

## Figures and Tables

**Table 1:** Participants' demographic and Work-Family Conflict characteristics.

Characteristics	Percentage	Mean ± SD	*p* value
Age			0.84
Up to 25	12.50	51 ± 12	
26–30	41.70	57 ± 10	
31–35	23.40	54 ± 10	
36–40	10.40	55 ± 11	
41–45	7.80	59 ± 13	
46 and higher	3.10	51 ± 8	

Sex			0.376
Male	97.40	55.7 ± 11	
Female	2.60	63 ± 26	

Marital status			0.950
Married	69.80	55.84 ± 11	
Single	30.2	55.98 ± 11	

Education			0.811
Up to junior high school	18.2	56.44 ± 11	
Associate degree	45.3	55.23 ± 11	
Bachelor	33.9	56.42 ± 11	
Master and higher	2.6	58.50 ± 3	

Family members			0.754
Up to 2	35	56.48 ± 7	
3–5	54.4	53.39 ± 8	
6 and higher	10	53.86 ± 7	

Need for care			0.329
Yes	51.8	56.87 ± 10	
No	48.2	54.94 ± 12	

Shift-work schedule			0.04
Daily (morning shift)	1.6	52.71 ± 7	
24-hour on/48-hour off	30.3	59.16 ± 13	
Variable	68.1	54.64 ± 10	

Employment status			0/05
Formal, governmental work	29.7	53.10 ± 9	
Nonformal, governmental work	9.4	56.83 ± 10	
Contractual, governmental work	54.2	57.50 ± 11	
2-year service commitment	6.7	49 ± 9	

Shift-work exchange limitation			0/02
Yes	34.9	58.6 ± 10	
No	63.5	56.16 ± 11	

Working hours per month			0.572
Obliged	7.9	57.75 ± 8	
Obliged and overtime	92.1	55.79 ± 11	

Workplace location			0.136
Rural EMS base	30.7	58.21 ± 11	
Urban EMS base	24.5	52.45 ± 8	
Rural and urban EMS base	38.5	56.6 ± 12	
Administrative unite/EOC	6.3	50.50 ± 2	

Workplace-home distance (km)			0.595
Up to 20	36.5	55.9 ± 9	
21–40	25.5	56.3 ± 12	
41–60	17.7	54.38 ± 12	
61 and higher	20.3	58.16 ± 12	

Training course			0.274
Yes	17.3	63.63 ± 13	
No	82.7	54.61 ± 10	

Household income			0.195
Very good	2.1	51.50 ± 14	
Good	9.4	55.00 ± 12	
Medline	73.4	55.00 ± 11	
Weak	14.1	61.26 ± 07	
Very weak	1	62.24 ± 10	

**Table 2:** Mean and standard deviation of Participants' Work-Family Conflict and time management skills.

Variable	Mean	Standard deviation
Total Work-Family Conflict	55.88	11.41
Work‐to‐Family Conflict	28.37	5.85
Family‐to‐Work Conflict	27.70	6.18
Total time management skills	122.14	16.69
Management of meetings	10.71	3.42
Management of communication	20.12	3.67
Operational planning	27.59	4.95
Set of goals	14.89	3.30
Prioritization of goals	19.36	3.74
Delegation of authority	21.67	3.72

**Table 3:** Correlation matrix, mean, and standard deviation based on Pearson correlation analysis.

Variable	1	2	3	4	Mean	Standard deviation
Tim management	1				122.14	16.69
Total Work-Family Conflict	0.383-^*∗∗*^	1			55.88	11.41
Work‐to‐Family Conflict	0.385-^*∗∗*^	0.950^*∗∗*^	1		28.37	5.85
Family‐to‐Work Conflict	0.342-^*∗∗*^	0.954^*∗∗*^	0.779^*∗∗*^	1	27.70	6.18

^*∗*^Significant at *p* < 0.05. ^*∗∗*^Significant at *p* < 0.001.

**Table 4:** Correlation, mean, and standard deviation based on Pearson correlation analysis.

Variable	Correlation with Work‐Family Conflict	Mean	Standard deviation
Management of meetings	0.025	10.71	3.42
Management of communication	0.054	20.12	3.67
Operational planning	^*∗∗*^−0.457	27.59	4.95
Set of goals	^*∗∗*^−0.27	14.89	3.30
Prioritization of goals	^*∗∗*^−0.345	19.36	3.74
Delegation of authority	^*∗∗*^−0.47	21.67	3.72

^*∗*^Significant at *p* < 0.05. ^*∗∗*^Significant at *p* < 0.001.

**Table 5:** Results of one-way analysis of variance (ANOVA) about relationship between total WFC score and personnel demographic factors.

Variable		Sum of squares	df	Mean	*F*	Standard deviation
Shift-work schedule	Between groups	548/577	1	54877	4/318	0/04
	Within group	16895/527	133	127/034		
	Total	17444/104	134			

Employment status	Between groups	1218/137	4	304/534	2/044	0/05
	Within group	16225/967	130	124/815		
	Total	17444/104	144			

Shift-work exchange restriction	Between groups	30/756	1	30.756	0/235	0/02
	Within group	17413/348	133	130.927		
	Total	17444/101	134			

## Data Availability

The datasets used and/or analyzed during the current study are available from the corresponding author on reasonable request.
